# Supplementary material for: The Relationship between Subjective Aging and Cognition in Elderly People: A Systematic Review
Source: Healthcare (Basel). 2023 Dec 7;11(24):3115. doi: 10.3390/healthcare11243115 (PMC10743019; doi:10.3390/healthcare11243115)
Supplement: Supplementary file 1 [file healthcare-11-03115-s001.zip › healthcare-2727907-supplementary/Supplementary data 1.docx]

**Supplementary data 1**

*Search terms and syntax*

## Search terms: Subjective aging

• Subjective aging.

• Age identity.

• Felt age.

• Desired age.

• Perceived age.

• Self-perceptions of aging.

• Perceptions of aging.

• Age beliefs.

• Aging perceptions.

• Views on aging.

• Personal experience of aging.

• Aging related cognitions.

• Aging satisfaction.

• Attitudes towards own aging.

• Attitudes to aging.

• Aging attitudes.

• Self-directed aging stereotypes.

• Aging stereotypes.

• Age stereotypes.

• Age-based stereotypes.

• Awareness of aging.

## Search strategy for PubMed

(“Subjective age” [TiAb:~2] OR “Subjective aging” [TiAb:~2] OR “Subjective ageing” [TiAb:~2] OR “age identity” [TiAb] OR “felt age” [TiAb] OR “desired age” [TiAb] OR “perceived age”[TiAb] OR “self-perceptions of aging” [TiAb:~2] OR “self-perception* of aging” [TiAb] OR “self-perceptions of ageing” [TiAb:~2] “self-perception* of ageing” [TiAb] OR “perceptions of aging” [TiAb:~2] OR “perception* of aging” [TiAb] OR “perceptions of ageing” [TiAb:~2] OR “perception* of ageing” [TiAb] OR “aging perceptions” [TiAb:~2] OR “aging perception*” [TiAb] OR “ageing perceptions” [TiAb:~2] OR “ageing perception*” [TiAb] OR “age perceptions” [TiAb:~2] OR “age perception*” [TiAb] OR “personal experience* of aging” [TiAb] OR “personal experience* of ageing” [TiAb] OR “personal experience of aging” [TiAb:~2] OR “personal experiences of aging” [TiAb:~2] OR “personal experience of ageing” [TiAb:~2] OR “personal experiences of ageing” [TiAb:~2] OR “aging related cognitions” [TiAb:~2] OR “ageing related cognitions” [TiAb:~2] OR “age related cognitions” [TiAb:~2] OR “aging cognitions” [TiAb:~2] OR “ageing cognitions” [TiAb:~2] OR “age cognitions” [TiAb:~2] OR “aging related cognition*” [TiAb] OR “ageing related cognition*” [TiAb] OR “age related cognition*” [TiAb] OR “aging cognition*” [TiAb] OR “ageing cognition*” [TiAb] OR “age cognition*” [TiAb] OR “aging satisfaction” [TiAb] OR “ageing satisfaction”[TiAb] OR “attitud* towards own aging” [TiAb] OR “attitud* towards own ageing”[TiAb] OR “attitudes towards own aging” [TiAb:~2] OR “attitudes towards own ageing”[TiAb:~2] OR “attitud* to aging”[TiAb] OR “attitude to aging”[TiAb:~2] OR “attitudes to aging”[TiAb:~2] OR “attitud* to ageing”[TiAb] OR “attitude to ageing”[TiAb:~2] OR “attitudes to ageing”[TiAb:~2] OR “aging attitude” [TiAb:~2] OR “aging attitudes” [TiAb:~2] OR “aging attitude*” [TiAb] OR “ageing attitude*” [TiAb] OR “ageing attitude” [TiAb:~2] OR “ageing attitudes” [TiAb:~2] OR “age attitud*” [TiAb] OR “age attitude” [TiAb:~2] OR “age attitudes” [TiAb:~2] OR “self-directed aging stereotyp*”[TiAb] OR “self-directed aging stereotype”[TiAb:~2] OR “self-directed aging stereotypes”[TiAb:~2] OR “self-directed ageing stereotypes”[TiAb:~2] OR “self-directed ageing stereotyp*”[TiAb] OR “self-directed ageing stereotype”[TiAb:~2] OR “aging stereotyp*”[TiAb] OR “aging stereotype”[TiAb:~2] OR “aging stereotypes”[TiAb:~2] OR “ageing stereotyp*”[TiAb] OR “ageing stereotype”[TiAb:~2] OR “ageing stereotypes”[TiAb:~2] OR “age stereotyp*”[TiAb] OR “age stereotype”[TiAb:~2] OR “age stereotypes”[TiAb:~2] OR “awareness of aging”[TiAb] OR "aging awareness"[TiAb:~2] OR "ageing awareness"[TiAb:~2] OR "age awareness"[TiAb:~2] OR “awareness of ageing”[TiAb] OR “view* on aging” [TiAb] OR “view* on ageing” [TiAb] OR "views on aging"[TiAb:~2] OR "views on ageing"[TiAb:~2] OR “age-based stereotyp*” [TiAb] OR “age-based stereotype” [TiAb:~2] OR “age-based stereotypes” [TiAb:~2] OR “age belie*"[TiAb] OR “age beliefs"[TiAb:~2]) AND (Attention[MeSH] OR attention [TiAb] OR age related memory disorder[MeSH] OR memory disorder[MeSH] OR episodic memory[MeSH] OR amnesia memory loss[MeSH] OR memory deficit[MeSH] OR episodic memory[MeSH] OR long term memory[MeSH] OR delayed memory[MeSH] OR memory [TiAb] OR executive function[MeSH] OR “executive funct*” [TiAb] OR “executive function” [TiAb:~2] OR “problem solv*” [TiAb] OR working memory[MeSH] OR “working memor*” [TiAb] OR “processing speed” [TiAb:~2] OR “speed of processing” [TiAb:~2] OR “process* speed” [TiAb] OR “speed of process*” [TiAb] OR “verbal fluency” [TiAb] OR verbal fluency disorder[MeSH] OR naming [TiAb] OR multitask* [TiAb] OR inhibit* [TiAb] OR inhibition, psychological[MeSH] OR interference [TiAb] OR distract* [TiAb] OR “task switch*”[TiAb] OR “task switch”[TiAb:~2] OR “task switching”[TiAb:~2] OR reason*[TiAb] OR plan*[TiAb] OR Intelligence[MeSH] OR intelligence [TiAb] OR executive control[MeSH] OR “executive control” [TiAb:~2] OR “cognitive control” [TiAb] OR cognition[MeSH] OR cognit* [TiAb] OR cognition disorder[MeSH] OR “cognitive decline” [TiAb:~2] OR “cognit* decline” [TiAb] OR “cognit* impairment” [TiAb] OR “cognitive impairment” [TiAb:~2] “cognitive aging” [TiAb:~2] OR “cognitive ageing” [TiAb:~2] OR dementia[MeSH] OR dementia [TiAb] OR alzheimer [TiAb])

## Search strategy for Psychinfo

TI "subjective age" OR AB "subjective age" OR KW "subjective age" OR TI "subjective aging" OR AB "subjective aging" OR KW "subjective aging" OR TI "subjective ageing" OR AB "subjective ageing" OR KW "subjective ageing" OR TI "felt age" OR AB "felt age" OR KW "felt age" OR TI "desired age" OR AB "desired age" OR KW "desired age" OR TI "perceived age" OR AB "perceived age" OR KW "perceived age" OR TI "self-perception* of aging" OR AB "self-perception* of aging" OR KW "self-perception* of aging" OR TI "aging perception*" OR AB "aging perception*" OR KW "aging perception*" OR TI "ageing perception*" OR AB "ageing perception*" OR KW "ageing perception*" OR TI "age perception*" OR AB "age perception*" OR KW "age perception*" OR TI "personal experience* of aging" OR AB "personal experience* of aging" OR KW "personal experience* of aging" OR TI "personal experience* of ageing" OR AB "personal experience* of ageing" OR KW "personal experience* of ageing" OR TI "aging related cognition*" OR AB "aging related cognition*" OR KW "aging related cognition*" OR TI "ageing related cognition*" OR AB "ageing related cognition*" OR KW "ageing related cognition*" OR TI "age related cognition*" OR AB "age related cognition*" OR KW "age related cognition*" OR TI "age cognition*" OR AB "age cognition*" OR KW "age cognition*" OR TI "aging cognition*" OR AB "aging cognition*" OR KW "aging cognition*"OR TI "ageing cognition*" OR AB "ageing cognition*" OR KW "ageing cognition*" OR TI "aging satisfaction" OR AB "aging satisfaction" OR KW "aging satisfaction" OR TI "ageing satisfaction" OR AB "ageing satisfaction" OR KW "ageing satisfaction" OR DE "Aging (Attitudes Toward)" OR TI "attitude* towards own aging" OR AB "attitude* towards own aging" OR KW "attitud* towards own aging" OR TI "attitud* towards own ageing" OR AB "attitud* towards own ageing" OR KW "attitud* towards own ageing" OR TI "attitud* to ageing" OR AB "attitude* to ageing" OR KW "attitud* to ageing" OR TI "attitud* to aging" OR AB "attitud* to aging" OR KW "attitud* to aging" OR TI "aging attitud*" OR AB "aging attitud*" OR KW "aging attitud*" OR TI "ageing attitud*" OR AB "ageing attitud*" OR KW "ageing attitud*" OR TI "age attitud*" OR AB "age attitud*" OR KW "age attitud*" OR TI "self-directed aging stereotyp*" OR AB "self-directed aging stereotyp*" OR KW "self-directed aging stereotyp*" TI "self-directed ageing stereotyp*" OR AB "self-directed ageing stereotyp*" OR KW "self-directed ageing stereotyp*" OR TI "aging stereotyp*" OR AB "aging stereotyp*" OR KW "aging stereotyp*" OR TI "ageing stereotyp*" OR AB "ageing stereotyp*" OR KW "ageing stereotyp*" OR TI "age stereotyp*" OR AB "age stereotyp*" OR KW "age stereotyp*" OR TI "awareness of aging" OR AB "awareness of aging" OR "KW awareness of aging" OR "TI awareness of ageing" OR AB "awareness of ageing" OR KW "awareness of ageing" OR KW "view* on aging" OR TI "view* on aging" OR AB "view* on aging" OR KW "view* on ageing" OR TI "view* on ageing" AB "view* on ageing" OR KW "age-based stereotyp*" OR TI "age-based stereotyp*" OR AB "age-based stereotyp*" OR TI "age belie*" OR AB ""age belie*" OR KW "age belie*"

AND

MA attention OR DE "Attention" OR TI attention OR AB attention OR KW attention OR MA age-related memory disorder OR MA memory disorder OR MA episodic memory OR MA amnesia memory loss OR MA memory deficit OR MA episodic memory OR MA long term memory OR MA delayed memory OR MA memory OR DE "Memory" OR DE ”Memory and Learning Measures” OR “DE “Memory Consolidation” OR DE “Memory Decay” OR DE “Memory Disorders” OR TI memory OR AB memory OR KW memory OR MA Executive function OR DE “Executive Function” OR DE “Cognitive Ability” OR TI "executive funct*" OR AB "executive funct*" OR KW "executive funct*" OR MA working memory OR DE “Short Term Memory” OR TI "working memory" OR AB "working memory" OR KW "working memory" OR TI "proces* speed" OR AB "process* speed" OR KW "proces* speed" OR TI "speed of process*" OR AB "speed of process*" OR KW "speed of process" OR DE ”Verbal Fluency” OR DE “Verbal Learning” OR DE “Verbal Memory” OR MA verbal fluency disorder OR TI "verbal fluency" OR AB "verbal fluency" OR KW "verbal fluency" OR DE “Naming” OR TI naming OR AB naming OR KW naming OR DE “Multitasking” OR TI multitask* OR AB multitask*OR KW multitask* OR MA inhibition, psychological OR DE “Proactive Inhibition” OR DE “Retroactive Inhibition” OR TI inhibit* OR AB inhibit* OR KW inhibit* OR DE “Interference (Learning)” OR TI interference OR AB interference OR KW interference OR DE “Distraction” OR TI distract* OR AB distract* OR KW distract* OR DE “Task Switching” OR TI "task switch*" OR AB "task switch*" OR KW "task switch*" OR DE “Reasoning” OR TI reason* OR AB reason* OR KW reason* OR TI plan* OR AB plan* OR KW plan* OR MA Intelligence OR DE “Intelligence” OR TI intelligence OR AB intelligence OR KW intelligence OR MA executive control OR TI "executive control" OR AB "executive control" OR KW "executive control" OR DE “Cognitive Control” OR TI "cognitive control" OR AB "cognitive control" OR KW "cognitive control" OR MA Cognition OR DE “Cognition” OR TI cognit* OR AB cognit* OR KW cognit* OR MA cognition disorder OR DE “Cognitive Aging” OR TI “Cognitive Aging” OR AB “Cognitive Aging” OR KW “Cognitive Aging” OR TI "cognitive decline" OR AB "cognitive decline" OR KW "cognitive decline" OR TI "cognitive disorder" OR AB "cognitive disorder" OR KW "cognitive disorder" OR TI "cognitive impairment" OR AB "cognitive impairment" OR KW "cognitive impairment" OR DE “Cognitive Impairment” OR DE “Mild Cognitive Impairment” OR MA Dementia OR DE “Dementia” OR DE “Dementia with Lewy Bodies” OR TI dementia OR AB dementia OR KW dementia OR TI Alzheimer OR AB alzheimer OR KW Alzheimer

## Search strategy for WoS

TI=(“subjective age”) OR AB=(“subjective age”) OR AK=(“subjective age”) OR TI=(“subjective aging”) OR AB=(“subjective aging”) OR AK=(“subjective aging”) OR TI=(“subjective ageing”) OR AB=(“subjective ageing”) OR AK=(“subjective ageing”) OR TI=(“felt age”) OR AB=(“felt age”) OR AK=(“felt age”) OR TI=(“desired age”) OR AB=(“desired age”) OR AK=(“desired age”) OR TI=(“perceived age”) OR AB=(“perceived age”) OR AK=(“perceived age”) OR TI=(“self-perceptions of aging”) OR AB=(“self-perceptions of aging”) OR AK=(“self-perceptions of aging”) OR TI=(“self-perceptions of ageing”) OR AB=(“self-perceptions of ageing”) OR AK=(“self-perceptions of ageing”) OR TI=(“perceptions of aging”) OR AB=(“perceptions of aging”) OR AK=(“perceptions of aging”) OR TI=(“perceptions of ageing”) OR AB=(“perceptions of ageing”) OR AK=(“perceptions of ageing”) OR TI=(“aging perceptions”) OR AB=(“aging perceptions”) OR AK=(“aging perceptions”) OR TI=(“ageing perceptions”) OR AB=(“ageing perceptions”) OR AK=(“ageing perceptions”) OR TI=(“age perceptions”) OR AB=(“age perceptions”) OR AK=(“age perceptions”) OR TI=(“personal experience* of aging”) OR AB=(“personal experience* of aging”) OR AK=(“personal experience* of aging”) OR TI=(“personal experience* of ageing”) OR AB=(“personal experience* of ageing”) OR AK=(“personal experience* of ageing”) OR TI=(“aging related cognitions”) OR AB=(“aging related cognitions”) OR AK=(“aging related cognitions”) OR TI=(“aging cognitions”) OR AB=(“aging cognitions”) OR AK=(“aging cognitions”) OR TI=(“ageing related cognitions”) OR AB=(“ageing related cognitions”) OR AK=(“ageing related cognitions”) OR TI=(“ageing cognitions”) OR AB=(“ageing cognitions”) OR AK=(“ageing cognitions”) OR TI=(“age related cognitions”) OR AB=(“age related cognitions”) OR AK=(“age related cognitions”) OR TI=(“age cognitions”) OR AB=(“age cognitions”) OR AK=(“age cognitions”) OR TI=(“aging satisfaction”) OR AB=(“aging satisfaction”) OR AK=(“aging satisfaction”) OR TI=(“ageing satisfaction”) OR AB=(“ageing satisfaction”) OR AK=(“ageing satisfaction”) OR TI=(“attitudes towards own aging”) OR AB=(“attitudes towards own aging”) OR AK=(“attitud* towards own aging”) OR TI=(“attitud* towards own ageing”) OR AB=(“attitud* towards own ageing”) OR AK=(“attitud* towards own ageing”) OR TI=(“attitud* to ageing”) OR AB=(“attitude* to ageing”) OR AK=(“attitud* to ageing”) OR TI=(“attitud* to aging”) OR AB=(“attitud* to aging”) OR AK=(“attitud* to aging”) OR TI=(“aging attitud*”) OR AB=(“aging attitud*”) OR AK=(“aging attitud*”) OR TI=(“ageing attitud*”) OR AB=(“ageing attitud*”) OR AK=(“ageing attitud*”) OR TI=(“age attitud*”) OR AB=(“age attitud*”) OR AK=(“age attitud*”) OR TI=(“self-directed aging stereotyp*”) OR AB=(“self-directed aging stereotyp*”) OR AK=(“self-directed aging stereotyp*”) OR TI=(“self-directed ageing stereotyp*”) OR AB=(“self-directed ageing stereotyp*”) OR AK=(“self-directed ageing stereotyp*”) OR TI=(“aging stereotyp*”) OR AB=(“aging stereotyp*”) OR AK=(“aging stereotyp*”) OR TI=(“ageing stereotyp*”) OR AB=(“ageing stereotyp*”) OR AK=(“ageing stereotyp*”) OR TI=(“age stereotyp*”) OR AB=(“age stereotyp*”) OR AK=(“age stereotyp*”) OR TI=(“awareness of aging”) OR AB=(“awareness of aging”) OR AK=(“awareness of aging”) OR TI=(“awareness of ageing”) OR AB=(“awareness of ageing”) OR AK=(“awareness of ageing”) OR AK=(“view* on aging”) OR TI=(“view* on aging”) OR AB=(“view* on aging”) OR AK=(“view* on ageing”) OR TI=(“view* on ageing”) OR AB=(“view* on ageing”) OR AK=(“ view* on ageing”) OR TI=(“age-based stereotyp*”) OR AB=(“age-based stereotyp*”) OR AK=(”age-based stereotyp*”) OR TI=(“age belie*”) OR AB=(“age belie*”) OR AK=(“age belie*”)

AND

TI=(attention) OR AB=(attention) OR AK=(attention) OR TI=(memory) OR AB=(memory) OR AK=(memory) OR TI=(“executive funct*”) OR AB=(“executive funct*”) OR AK=(“executive funct*”) OR TI=(“working memory”) OR AB=(“working memory”) OR AK=(“working memory”) OR TI=(“processing speed”) OR AB=(“processing speed”) OR AK=(“processing speed”) OR TI=(“speed of processing”) OR AB=(“speed of processing”) OR AK=(“speed of processing”) OR TI=(“verbal fluency”) OR AB=(“verbal fluency”) OR AK=(“verbal fluency”) OR TI=(naming) OR AB=(naming) OR AK=(naming) OR TI=(“multitask*”) OR AB=(“multitask*”) OR AK=(“multitask*”) OR TI=(inhibit*) OR AB=(inhibit*) OR AK=(inhibit*) OR TI=(interference) OR AB=(interference) OR AK=(interference) OR TI=(distract*) OR AB=(distract*) OR AK=(distract*) OR TI=(“task switch*”) OR AB=(“task switch*”) OR AK=(“task switch*”) OR TI=(reason*) OR AB=(reason*) OR AK=(reason*) OR TI=(plan*) OR AB=(plan*) OR AK=(plan*) OR TI=(intelligence) OR AB=(intelligence) OR AK=(intelligence) OR TI=(“executive control”) OR AB=(“executive control”) OR AK=(“executive control”) OR TI=(“cognitive control”) OR AB=(“cognitive control”) OR AK=(“cognitive control”) OR TI=(cognit*) OR AB=(cognit*) OR AK=(cognit*) OR TI=(“cognitive decline”) OR AB=(“cognitive decline”) OR AK=(“cognitive decline”) OR TI=(“cognitive disorder”) OR AB=(“cognitive disorder”) OR AK=(“cognitive disorder”) OR TI=(“cognitive impairment”) OR AB=(“cognitive impairment”) OR AK=(“cognitive impairment”) OR TI=(“cognitive aging”) OR AB=(“cognitive aging”) OR AK=(“cognitive aging”) OR TI=(dementia) OR AB=(dementia) OR AK=(dementia) OR TI=(Alzheimer)OR AB=(Alzheimer) OR AK=(Alzheimer)

## Search strategy for Google Scholar (Allintitle)

Search 1

“Subjective age” AND (Attention|Memory|Executive|Cognition| |Process|Fluency|Naming|Multitask|Inhibition|Interference|Reason|Intelligence|Plan|Dementia|“cognitive decline”|“Mild cognitive impairment”)

Search 2

“Subjective aging” AND (Attention|Memory|Executive|Cognition| |Process|Fluency|Naming|Multitask|Inhibition|Interference|Reason|Intelligence|Plan|Dementia|“cognitive decline”|“Mild cognitive impairment”)

Search 3

“Subjective ageing” AND (Attention|Memory|Executive|Cognition| |Process|Fluency|Naming|Multitask|Inhibition|Interference|Reason|Intelligence|Plan|Dementia|“cognitive decline”|“Mild cognitive impairment”)

Search 4

“Felt age” AND (Attention|Memory|Executive|Cognition| |Process|Fluency|Naming|Multitask|Inhibition|Interference|Reason|Intelligence|Plan|Dementia|“cognitive decline”|“Mild cognitive impairment”)

Search 5

“Desired age” AND (Attention|Memory|Executive|Cognition| |Process|Fluency|Naming|Multitask|Inhibition|Interference|Reason|Intelligence|Plan|Dementia|“cognitive decline”|“Mild cognitive impairment”)

Search 6

“Perceived age” AND (Attention|Memory|Executive|Cognition| |Process|Fluency|Naming|Multitask|Inhibition|Interference|Reason|Intelligence|Plan|Dementia|“cognitive decline”|“Mild cognitive impairment”)

Search 7

“Self-perceptions of aging” AND (Attention|Memory|Executive|Cognition| |Process|Fluency|Naming|Multitask|Inhibition|Interference|Reason|Intelligence|Plan|Dementia|“cognitive decline”|“Mild cognitive impairment”)

Search 8

“Self-perceptions of ageing” AND (Attention|Memory|Executive|Cognition| |Process|Fluency|Naming|Multitask|Inhibition|Interference|Reason|Intelligence|Plan|Dementia|“cognitive decline”|“Mild cognitive impairment”)

Search 9

“Aging perceptions” AND (Attention|Memory|Executive|Cognition| |Process|Fluency|Naming|Multitask|Inhibition|Interference|Reason|Intelligence|Plan|Dementia|“cognitive decline”|“Mild cognitive impairment”)

Search 10

“Aging perception” AND (Attention|Memory|Executive|Cognition| |Process|Fluency|Naming|Multitask|Inhibition|Interference|Reason|Intelligence|Plan|Dementia|“cognitive decline”|“Mild cognitive impairment”)

Search 11

“Ageing perceptions” AND (Attention|Memory|Executive|Cognition| |Process|Fluency|Naming|Multitask|Inhibition|Interference|Reason|Intelligence|Plan|Dementia|“cognitive decline”|“Mild cognitive impairment”)

Search 12

“Ageing perception” AND (Attention|Memory|Executive|Cognition| |Process|Fluency|Naming|Multitask|Inhibition|Interference|Reason|Intelligence|Plan|Dementia|“cognitive decline”|“Mild cognitive impairment”)

Search 13

“Personal experience of aging” AND (Attention|Memory|Executive|Cognition| |Process|Fluency|Naming|Multitask|Inhibition|Interference|Reason|Intelligence|Plan|Dementia|“cognitive decline”|“Mild cognitive impairment”)

Search 14

“Personal experience of ageing” AND (Attention|Memory|Executive|Cognition| |Process|Fluency|Naming|Multitask|Inhibition|Interference|Reason|Intelligence|Plan|Dementia|“cognitive decline”|“Mild cognitive impairment”)

Search 15

“Personal experiences of ageing” AND (Attention|Memory|Executive|Cognition| |Process|Fluency|Naming|Multitask|Inhibition|Interference|Reason|Intelligence|Plan|Dementia|“cognitive decline”|“Mild cognitive impairment”)

Search 16

“Personal experiences of aging” AND (Attention|Memory|Executive|Cognition| |Process|Fluency|Naming|Multitask|Inhibition|Interference|Reason|Intelligence|Plan|Dementia|“cognitive decline”|“Mild cognitive impairment”)

Search 17

“Aging-related cognitions” AND (Attention|Memory|Executive|Cognition| |Process|Fluency|Naming|Multitask|Inhibition|Interference|Reason|Intelligence|Plan|Dementia|“cognitive decline”|“Mild cognitive impairment”)

Search 18

“Ageing-related cognitions” AND (Attention|Memory|Executive|Cognition| |Process|Fluency|Naming|Multitask|Inhibition|Interference|Reason|Intelligence|Plan|Dementia|“cognitive decline”|“Mild cognitive impairment”)

Search 19

“Age-related cognitions” AND (Attention|Memory|Executive|Cognition| |Process|Fluency|Naming|Multitask|Inhibition|Interference|Reason|Intelligence|Plan|Dementia|“cognitive decline”|“Mild cognitive impairment”)

Search 20

“Aging satisfaction” AND (Attention|Memory|Executive|Cognition| |Process|Fluency|Naming|Multitask|Inhibition|Interference|Reason|Intelligence|Plan|Dementia|“cognitive decline”|“Mild cognitive impairment”)

Search 21

“Ageing satisfaction” AND (Attention|Memory|Executive|Cognition| |Process|Fluency|Naming|Multitask|Inhibition|Interference|Reason|Intelligence|Plan|Dementia|“cognitive decline”|“Mild cognitive impairment”)

Search 22

“Attitudes towards own aging” AND (Attention|Memory|Executive|Cognition| |Process|Fluency|Naming|Multitask|Inhibition|Interference|Reason|Intelligence|Plan|Dementia|“cognitive decline”|“Mild cognitive impairment”)

Search 23

“Attitudes towards own ageing” AND (Attention|Memory|Executive|Cognition| |Process|Fluency|Naming|Multitask|Inhibition|Interference|Reason|Intelligence|Plan|Dementia|“cognitive decline”|“Mild cognitive impairment”)

Search 24

“Aging attitudes” AND (Attention|Memory|Executive|Cognition| |Process|Fluency|Naming|Multitask|Inhibition|Interference|Reason|Intelligence|Plan|Dementia|“cognitive decline”|“Mild cognitive impairment”)

Search 25

“Ageing attitudes” AND (Attention|Memory|Executive|Cognition| |Process|Fluency|Naming|Multitask|Inhibition|Interference|Reason|Intelligence|Plan|Dementia|“cognitive decline”|“Mild cognitive impairment”)

Search 26

“Attitudes to aging” AND (Attention|Memory|Executive|Cognition| |Process|Fluency|Naming|Multitask|Inhibition|Interference|Reason|Intelligence|Plan|Dementia|“cognitive decline”|“Mild cognitive impairment”)

Search 27

“Attitudes to ageing” AND (Attention|Memory|Executive|Cognition| |Process|Fluency|Naming|Multitask|Inhibition|Interference|Reason|Intelligence|Plan|Dementia|“cognitive decline”|“Mild cognitive impairment”)

Search 28

“Age attitudes” AND (Attention|Memory|Executive|Cognition| |Process|Fluency|Naming|Multitask|Inhibition|Interference|Reason|Intelligence|Plan|Dementia|“cognitive decline”|“Mild cognitive impairment”)

Search 29

“Self-directed aging stereotypes” AND (Attention|Memory|Executive|Cognition| |Process|Fluency|Naming|Multitask|Inhibition|Interference|Reason|Intelligence|Plan|Dementia|“cognitive decline”|“Mild cognitive impairment”)

Search 30

“Self-directed ageing stereotypes” AND (Attention|Memory|Executive|Cognition| |Process|Fluency|Naming|Multitask|Inhibition|Interference|Reason|Intelligence|Plan|Dementia|“cognitive decline”|“Mild cognitive impairment”)

Search 31

“Aging stereotypes” AND (Attention|Memory|Executive|Cognition| |Process|Fluency|Naming|Multitask|Inhibition|Interference|Reason|Intelligence|Plan|Dementia|“cognitive decline”|“Mild cognitive impairment”)

Search 32

“Ageing stereotypes” AND (Attention|Memory|Executive|Cognition| |Process|Fluency|Naming|Multitask|Inhibition|Interference|Reason|Intelligence|Plan|Dementia|“cognitive decline”|“Mild cognitive impairment”)

Search 33

“Age stereotypes” AND (Attention|Memory|Executive|Cognition| |Process|Fluency|Naming|Multitask|Inhibition|Interference|Reason|Intelligence|Plan|Dementia|“cognitive decline”|“Mild cognitive impairment”)

Search 34

“Age-based stereotypes” AND (Attention|Memory|Executive|Cognition| |Process|Fluency|Naming|Multitask|Inhibition|Interference|Reason|Intelligence|Plan|Dementia|“cognitive decline”|“Mild cognitive impairment”)

Search 35

“Awareness of aging” AND (Attention|Memory|Executive|Cognition| |Process|Fluency|Naming|Multitask|Inhibition|Interference|Reason|Intelligence|Plan|Dementia|“cognitive decline”|“Mild cognitive impairment”)

Search 36

“Awareness of ageing” AND (Attention|Memory|Executive|Cognition| |Process|Fluency|Naming|Multitask|Inhibition|Interference|Reason|Intelligence|Plan|Dementia|“cognitive decline”|“Mild cognitive impairment”)

Search 37

“Views on aging” AND (Attention|Memory|Executive|Cognition| |Process|Fluency|Naming|Multitask|Inhibition|Interference|Reason|Intelligence|Plan|Dementia|“cognitive decline”|“Mild cognitive impairment”)

Search 38

“Views on ageing” AND (Attention|Memory|Executive|Cognition| |Process|Fluency|Naming|Multitask|Inhibition|Interference|Reason|Intelligence|Plan|Dementia|“cognitive decline”|“Mild cognitive impairment”)

Search 39

“Perceptions of aging” AND (Attention|Memory|Executive|Cognition| |Process|Fluency|Naming|Multitask|Inhibition|Interference|Reason|Intelligence|Plan|Dementia|“cognitive decline”|“Mild cognitive impairment”)

Search 40

“Perceptions of ageing” AND (Attention|Memory|Executive|Cognition| |Process|Fluency|Naming|Multitask|Inhibition|Interference|Reason|Intelligence|Plan|Dementia|“cognitive decline”|“Mild cognitive impairment”)

Search 41

“Age beliefs” AND (Attention|Memory|Executive|Cognition| |Process|Fluency|Naming|Multitask|Inhibition|Interference|Reason|Intelligence|Plan|Dementia|“cognitive decline”|“Mild cognitive impairment”)

## Search strategy for WorldCat (Dissertation/Thesis)

Search 1:

(kw:"Subjective age" OR kw:"Subjective aging" OR kw:"Subjective ageing" OR kw:"age identity" OR kw:"felt age" OR kw:"desired age" OR kw:"perceived age" OR kw:"self-perception* of aging" OR kw:"self-perception* of ageing" OR kw:"aging perception*" OR kw:"ageing perception*" OR kw:"age perception*" OR kw:"personal experienc* of aging" OR kw:"personal experience* of ageing" OR kw:"aging related cognition*" OR kw:"ageing related cognition*" OR kw:"age related cognition*" OR kw:"aging satisfaction" OR kw:"ageing satisfaction" OR kw:"attitud* towards own aging" OR kw:"attitud* towards own ageing" OR kw:"attitud* to aging" OR kw:"attitud* to ageing" OR kw:"aging attitud*" OR kw:"ageing attitud*" OR kw:"age attitud*" OR kw:"self-directed aging stereotyp*" OR kw:"self-directed ageing stereotyp*" OR kw:"aging stereotyp*" OR kw:"ageing stereotyp*" OR kw:"age stereotyp*" OR kw:"awareness of aging" OR kw:"awareness of ageing" OR kw:"view* on aging" OR kw:"view* on ageing" OR kw:"age belie*" OR kw:"perception* of aging" OR kw:"perception* of ageing") AND (kw:Attention OR kw:Memory OR kw:"executive funct*" OR kw:"problem solv*" OR kw:"working memor*" OR kw:"process* speed" OR kw:"speed of process*" OR kw:"verbal fluency" OR kw:naming OR kw:"multitask*" OR kw:inhibit* OR kw:interferenc* OR kw:distract* OR kw:"task switch*" OR kw:reason* OR kw:plan* OR kw:Intelligence OR kw:"executive control" OR kw:"cognitive control*" OR kw:cognit* OR kw:"cognition disorder" OR kw:"cognitive decline" OR kw:"cognitive impairment" OR kw:dementia OR kw:Alzheimer* OR kw:Parkinson* OR kw:Lewy* OR kw:Corticobasal OR kw:"primary progressive aphasia" OR kw:neurodege*)

Search 2

(ti:"Subjective age" OR ti:"Subjective aging" OR ti:"Subjective ageing" OR ti:"age identity" OR ti:"felt age" OR ti:"desired age" OR ti:"perceived age" OR ti:"self-perception* of aging" OR ti:"self-perception* of ageing" OR ti:"aging perception*" OR ti:"ageing perception*" OR ti:"age perception*" OR ti:"personal experienc* of aging" OR ti:"personal experience* of ageing" OR ti:"aging related cognition*" OR ti:"ageing related cognition*" OR ti:"age related cognition*" OR ti:"aging satisfaction" OR ti:"ageing satisfaction" OR ti:"attitud* towards own aging" OR ti:"attitud* towards own ageing" OR ti:"attitud* to aging" OR ti:"attitud* to ageing" OR ti:"aging attitud*" OR ti:"ageing attitud*" OR ti:"age attitud*" OR ti:"self-directed aging stereotyp*" OR ti:"self-directed ageing stereotyp*" OR ti:"aging stereotyp*" OR ti:"ageing stereotyp*" OR ti:"age stereotyp*" OR ti:"awareness of aging" OR ti:"awareness of ageing" OR ti:"view* on aging" OR ti:"view* on ageing" OR ti:"age belie*" OR ti:"perception* of aging" OR ti:"perception* of ageing") AND (ti:Attention OR ti:Memory OR ti:"executive funct*" OR ti:"problem solv*" OR ti:"working memor*" OR ti:"process* speed" OR ti:"speed of process*" OR ti:"verbal fluency" OR ti:naming OR ti:"multitask*" OR ti:inhibit* OR ti:interferenc* OR ti:distract* OR ti:"task switch*" OR ti:reason* OR ti:plan* OR ti:Intelligence OR ti:"executive control" OR ti:"cognitive control*" OR ti:cognit* OR ti:"cognition disorder" OR ti:"cognitive decline" OR ti:"cognitive impairment" OR ti:dementia OR ti:Alzheimer* OR ti:Parkinson* OR ti:Lewy* OR ti:Corticobasal OR ti:"primary progressive aphasia" OR ti:neurodege*)

## Search strategy for OpenGrey

("Subjective age" OR "Subjective aging" OR "Subjective ageing" OR "age identity" OR "felt age" OR "desired age" OR "perceived age" OR "self-perception* of aging" OR "self-perception* of ageing" OR "aging perception*" OR "ageing perception*" OR "age perception*" OR "personal experienc* of aging" OR "personal experience* of ageing" OR "aging related cognition*" OR "ageing related cognition*" OR "age related cognition*" OR "aging satisfaction" OR "ageing satisfaction" OR "attitud* towards own aging" OR "attitud* towards own ageing" OR "attitud* to aging" OR "attitud* to ageing" OR "aging attitud*" OR "ageing attitud*" OR "age attitud*" OR "self-directed aging stereotyp*" OR "self-directed ageing stereotyp*" OR "aging stereotyp*" OR "ageing stereotyp*" OR "age stereotyp*" OR "awareness of aging" OR "awareness of ageing" OR "view* on aging" OR "view* on ageing")

## Search strategy for NDLTD

Search 1

(title:"subjective age" OR subject:"subjective age" OR title:"subjective aging" OR subject:"subjective aging" OR title:"subjective ageing" OR subject:"subjective ageing" OR title:"felt age" OR subject:"felt age" OR title:"desired age" OR subject:"desired age" OR title:"perceived age" OR subject:"perceived age" OR description:"perceived age" OR title:"self perceptions of aging" OR subject:"self perceptions of aging" OR description:"self perceptions of aging" OR title:"self perceptions of ageing" OR subject:"self perceptions of ageing" OR description:"self perceptions of ageing" OR title:"perceptions of aging" OR subject:"perceptions of aging" OR description:"perceptions of aging" OR title:"perceptions of ageing" OR subject:"perceptions of ageing" OR description:"perceptions of ageing" OR title:"aging perceptions" OR subject:"aging perceptions" OR description:"aging perceptions" OR title:"ageing perceptions" OR subject:"ageing perceptions" OR description:"ageing perceptions" OR title:"age perceptions" OR subject:"age perceptions" OR description:"age perceptions" OR title:"personal experience of aging" OR subject:"personal experience of aging" OR description:"personal experience of aging" OR title:"personal experiences of aging" OR subject:"personal experiences of aging" OR description:"personal experiences of aging" OR title:"personal experience of ageing" OR subject:"personal experience of ageing" OR description:"personal experience of ageing" OR title:"personal experiences of ageing" OR subject:"personal experiences of ageing" OR description:"personal experiences of ageing"title:"aging related cognitions" OR subject:"aging related cognitions" OR description:"aging related cognitions" OR title:"aging cognitions" OR subject:"aging cognitions" OR description:"aging cognitions" OR title:"ageing related cognitions" OR subject:"ageing related cognitions" OR description:"ageing related cognitions" OR title:"ageing cognitions" OR subject:"ageing cognitions" OR description:"ageing cognitions" OR title:"age related cognitions" OR subject:"age related cognitions" OR description:"age related cognitions" OR title:"age cognitions" OR subject:"age cognitions" OR description:"age cognitions" OR title:"aging satisfaction" OR subject:"aging satisfaction" OR description:"aging satisfaction" OR title:"ageing satisfaction" OR subject:"ageing satisfaction" OR description:"ageing satisfaction" OR title:"attitudes towards own aging" OR subject:"attitudes towards own aging" OR description:"attitudes towards own aging" OR title:"attitudes towards own ageing" OR subject:"attitudes towards own ageing" OR description:"attitudes towards own ageing" OR title:"attitudes to ageing" OR subject:"attitudes to ageing" OR description:"attitud* to ageing" OR title:"attitudes to aging" OR subject:"attitudes to aging" OR description:"attitudes to aging" OR title:"aging attitudes" OR subject:"aging attitudes" OR description:"aging attitudes" OR title:"ageing attitudes" OR subject:"ageing attitudes" OR description:"ageing attitudes" OR title:"age attitudes" OR subject:"age attitudes" OR description:"age attitudes" OR title:"self directed aging stereotypes" OR subject:"self directed aging stereotypes" OR description:"self directed aging stereotypes" OR title:"self directed ageing stereotypes" OR subject:"self directed ageing stereotypes" OR description:"self directed ageing stereotypes" OR title:"aging stereotypes" OR subject:"aging stereotypes" OR description:"aging stereotypes" OR title:"ageing stereotypes" OR subject:"ageing stereotypes" OR description:"ageing stereotypes" OR title:"age stereotypes" OR subject:"age stereotypes" OR description:"age stereotypes" OR title:"age stereotype" OR subject:"age stereotype" OR description:"age stereotype"OR title:"awareness of aging" OR subject:"awareness of aging" OR description:"awareness of aging" OR title:"awareness of ageing" OR subject:"awareness of ageing" OR description:"awareness of ageing" OR description:"view on aging" OR title:"view on aging" OR subject:"view on aging" OR description:"views on aging" OR title:"views on aging" OR subject:"views on aging" OR description:"view on ageing" OR title:"view on ageing" OR subject:"view on ageing" OR description:"views on ageing" OR title:"views on ageing" OR subject:"views on ageing"title:"age based stereotypes" OR subject:"age based stereotypes" OR description:"age based stereotypes" OR title:"age beliefs" OR subject:"age beliefs" OR description:"age beliefs") AND (title: "attention" OR subject:"attention " OR description:"attention" OR title:"memory" OR subject:"memory" OR description:"memory" OR title:"executive funct*" OR subject:"executive funct*" OR description:"executive function" OR title:"working memory" OR subject:"working memory" OR description:"working memory" OR title:"processing speed" OR subject:"processing speed" OR description:"processing speed" OR title:"speed of processing" OR subject:"speed of processing" OR description:"speed of processing")

Search 2

(title:"subjective age" OR subject:"subjective age" OR title:"subjective aging" OR subject:"subjective aging" OR title:"subjective ageing" OR subject:"subjective ageing" OR title:"felt age" OR subject:"felt age" OR title:"desired age" OR subject:"desired age" OR title:"perceived age" OR subject:"perceived age" OR description:"perceived age" OR title:"self perceptions of aging" OR subject:"self perceptions of aging" OR description:"self perceptions of aging" OR title:"self perceptions of ageing" OR subject:"self perceptions of ageing" OR description:"self perceptions of ageing" OR title:"perceptions of aging" OR subject:"perceptions of aging" OR description:"perceptions of aging" OR title:"perceptions of ageing" OR subject:"perceptions of ageing" OR description:"perceptions of ageing" OR title:"aging perceptions" OR subject:"aging perceptions" OR description:"aging perceptions" OR title:"ageing perceptions" OR subject:"ageing perceptions" OR description:"ageing perceptions" OR title:"age perceptions" OR subject:"age perceptions" OR description:"age perceptions" OR title:"personal experience of aging" OR subject:"personal experience of aging" OR description:"personal experience of aging" OR title:"personal experiences of aging" OR subject:"personal experiences of aging" OR description:"personal experiences of aging" OR title:"personal experience of ageing" OR subject:"personal experience of ageing" OR description:"personal experience of ageing" OR title:"personal experiences of ageing" OR subject:"personal experiences of ageing" OR description:"personal experiences of ageing"title:"aging related cognitions" OR subject:"aging related cognitions" OR description:"aging related cognitions" OR title:"aging cognitions" OR subject:"aging cognitions" OR description:"aging cognitions" OR title:"ageing related cognitions" OR subject:"ageing related cognitions" OR description:"ageing related cognitions" OR title:"ageing cognitions" OR subject:"ageing cognitions" OR description:"ageing cognitions" OR title:"age related cognitions" OR subject:"age related cognitions" OR description:"age related cognitions" OR title:"age cognitions" OR subject:"age cognitions" OR description:"age cognitions" OR title:"aging satisfaction" OR subject:"aging satisfaction" OR description:"aging satisfaction" OR title:"ageing satisfaction" OR subject:"ageing satisfaction" OR description:"ageing satisfaction" OR title:"attitudes towards own aging" OR subject:"attitudes towards own aging" OR description:"attitudes towards own aging" OR title:"attitudes towards own ageing" OR subject:"attitudes towards own ageing" OR description:"attitudes towards own ageing" OR title:"attitudes to ageing" OR subject:"attitudes to ageing" OR description:"attitud* to ageing" OR title:"attitudes to aging" OR subject:"attitudes to aging" OR description:"attitudes to aging" OR title:"aging attitudes" OR subject:"aging attitudes" OR description:"aging attitudes" OR title:"ageing attitudes" OR subject:"ageing attitudes" OR description:"ageing attitudes" OR title:"age attitudes" OR subject:"age attitudes" OR description:"age attitudes" OR title:"self directed aging stereotypes" OR subject:"self directed aging stereotypes" OR description:"self directed aging stereotypes" OR title:"self directed ageing stereotypes" OR subject:"self directed ageing stereotypes" OR description:"self directed ageing stereotypes" OR title:"aging stereotypes" OR subject:"aging stereotypes" OR description:"aging stereotypes" OR title:"ageing stereotypes" OR subject:"ageing stereotypes" OR description:"ageing stereotypes" OR title:"age stereotypes" OR subject:"age stereotypes" OR description:"age stereotypes" OR title:"age stereotype" OR subject:"age stereotype" OR description:"age stereotype"OR title:"awareness of aging" OR subject:"awareness of aging" OR description:"awareness of aging" OR title:"awareness of ageing" OR subject:"awareness of ageing" OR description:"awareness of ageing" OR description:"view on aging" OR title:"view on aging" OR subject:"view on aging" OR description:"views on aging" OR title:"views on aging" OR subject:"views on aging" OR description:"view on ageing" OR title:"view on ageing" OR subject:"view on ageing" OR description:"views on ageing" OR title:"views on ageing" OR subject:"views on ageing"title:"age based stereotypes" OR subject:"age based stereotypes" OR description:"age based stereotypes" OR title:"age beliefs" OR subject:"age beliefs" OR description:"age beliefs") AND (title:"verbal fluency" OR subject:"verbal fluency" OR description:"verbal fluency" OR title:naming OR subject:"naming" OR description:"naming" OR title:"multitask" OR subject:"multitask" OR description:"multitask" OR title:"multitasking" OR subject:"multitasking" OR description:"multitasking" OR title:"inhibit" OR subject:"inhibit" OR description:"inhibit" OR title:"interference" OR subject:"interference" OR description:"interference" OR title:"distraction" OR subject:"distraction" OR description:"distraction")

Search 3

(title:"subjective age" OR subject:"subjective age" OR title:"subjective aging" OR subject:"subjective aging" OR title:"subjective ageing" OR subject:"subjective ageing" OR title:"felt age" OR subject:"felt age" OR title:"desired age" OR subject:"desired age" OR title:"perceived age" OR subject:"perceived age" OR description:"perceived age" OR title:"self perceptions of aging" OR subject:"self perceptions of aging" OR description:"self perceptions of aging" OR title:"self perceptions of ageing" OR subject:"self perceptions of ageing" OR description:"self perceptions of ageing" OR title:"perceptions of aging" OR subject:"perceptions of aging" OR description:"perceptions of aging" OR title:"perceptions of ageing" OR subject:"perceptions of ageing" OR description:"perceptions of ageing" OR title:"aging perceptions" OR subject:"aging perceptions" OR description:"aging perceptions" OR title:"ageing perceptions" OR subject:"ageing perceptions" OR description:"ageing perceptions" OR title:"age perceptions" OR subject:"age perceptions" OR description:"age perceptions" OR title:"personal experience of aging" OR subject:"personal experience of aging" OR description:"personal experience of aging" OR title:"personal experiences of aging" OR subject:"personal experiences of aging" OR description:"personal experiences of aging" OR title:"personal experience of ageing" OR subject:"personal experience of ageing" OR description:"personal experience of ageing" OR title:"personal experiences of ageing" OR subject:"personal experiences of ageing" OR description:"personal experiences of ageing"title:"aging related cognitions" OR subject:"aging related cognitions" OR description:"aging related cognitions" OR title:"aging cognitions" OR subject:"aging cognitions" OR description:"aging cognitions" OR title:"ageing related cognitions" OR subject:"ageing related cognitions" OR description:"ageing related cognitions" OR title:"ageing cognitions" OR subject:"ageing cognitions" OR description:"ageing cognitions" OR title:"age related cognitions" OR subject:"age related cognitions" OR description:"age related cognitions" OR title:"age cognitions" OR subject:"age cognitions" OR description:"age cognitions" OR title:"aging satisfaction" OR subject:"aging satisfaction" OR description:"aging satisfaction" OR title:"ageing satisfaction" OR subject:"ageing satisfaction" OR description:"ageing satisfaction" OR title:"attitudes towards own aging" OR subject:"attitudes towards own aging" OR description:"attitudes towards own aging" OR title:"attitudes towards own ageing" OR subject:"attitudes towards own ageing" OR description:"attitudes towards own ageing" OR title:"attitudes to ageing" OR subject:"attitudes to ageing" OR description:"attitud* to ageing" OR title:"attitudes to aging" OR subject:"attitudes to aging" OR description:"attitudes to aging" OR title:"aging attitudes" OR subject:"aging attitudes" OR description:"aging attitudes" OR title:"ageing attitudes" OR subject:"ageing attitudes" OR description:"ageing attitudes" OR title:"age attitudes" OR subject:"age attitudes" OR description:"age attitudes" OR title:"self directed aging stereotypes" OR subject:"self directed aging stereotypes" OR description:"self directed aging stereotypes" OR title:"self directed ageing stereotypes" OR subject:"self directed ageing stereotypes" OR description:"self directed ageing stereotypes" OR title:"aging stereotypes" OR subject:"aging stereotypes" OR description:"aging stereotypes" OR title:"ageing stereotypes" OR subject:"ageing stereotypes" OR description:"ageing stereotypes" OR title:"age stereotypes" OR subject:"age stereotypes" OR description:"age stereotypes" OR title:"age stereotype" OR subject:"age stereotype" OR description:"age stereotype"OR title:"awareness of aging" OR subject:"awareness of aging" OR description:"awareness of aging" OR title:"awareness of ageing" OR subject:"awareness of ageing" OR description:"awareness of ageing" OR description:"view on aging" OR title:"view on aging" OR subject:"view on aging" OR description:"views on aging" OR title:"views on aging" OR subject:"views on aging" OR description:"view on ageing" OR title:"view on ageing" OR subject:"view on ageing" OR description:"views on ageing" OR title:"views on ageing" OR subject:"views on ageing"title:"age based stereotypes" OR subject:"age based stereotypes" OR description:"age based stereotypes" OR title:"age beliefs" OR subject:"age beliefs" OR description:"age beliefs") AND (title:"task switch" OR subject:"task switch" OR description:"task switch" OR title:"task switching" OR subject:"task switching" OR description:"task switching" OR title:"reasoning" OR subject:"reasoning" OR description:"reasoning" OR title:"planning" OR subject:"planning" OR description:"planning" OR title:"planification" OR subject:"planification" OR description:"planification" OR title:"intelligence" OR subject:"intelligence" OR description:"intelligence" OR title:"executive control" OR subject:"executive control" OR description:"executive control")

Search 4

(title:"subjective age" OR subject:"subjective age" OR title:"subjective aging" OR subject:"subjective aging" OR title:"subjective ageing" OR subject:"subjective ageing" OR title:"felt age" OR subject:"felt age" OR title:"desired age" OR subject:"desired age" OR title:"perceived age" OR subject:"perceived age" OR description:"perceived age" OR title:"self perceptions of aging" OR subject:"self perceptions of aging" OR description:"self perceptions of aging" OR title:"self perceptions of ageing" OR subject:"self perceptions of ageing" OR description:"self perceptions of ageing" OR title:"perceptions of aging" OR subject:"perceptions of aging" OR description:"perceptions of aging" OR title:"perceptions of ageing" OR subject:"perceptions of ageing" OR description:"perceptions of ageing" OR title:"aging perceptions" OR subject:"aging perceptions" OR description:"aging perceptions" OR title:"ageing perceptions" OR subject:"ageing perceptions" OR description:"ageing perceptions" OR title:"age perceptions" OR subject:"age perceptions" OR description:"age perceptions" OR title:"personal experience of aging" OR subject:"personal experience of aging" OR description:"personal experience of aging" OR title:"personal experiences of aging" OR subject:"personal experiences of aging" OR description:"personal experiences of aging" OR title:"personal experience of ageing" OR subject:"personal experience of ageing" OR description:"personal experience of ageing" OR title:"personal experiences of ageing" OR subject:"personal experiences of ageing" OR description:"personal experiences of ageing"title:"aging related cognitions" OR subject:"aging related cognitions" OR description:"aging related cognitions" OR title:"aging cognitions" OR subject:"aging cognitions" OR description:"aging cognitions" OR title:"ageing related cognitions" OR subject:"ageing related cognitions" OR description:"ageing related cognitions" OR title:"ageing cognitions" OR subject:"ageing cognitions" OR description:"ageing cognitions" OR title:"age related cognitions" OR subject:"age related cognitions" OR description:"age related cognitions" OR title:"age cognitions" OR subject:"age cognitions" OR description:"age cognitions" OR title:"aging satisfaction" OR subject:"aging satisfaction" OR description:"aging satisfaction" OR title:"ageing satisfaction" OR subject:"ageing satisfaction" OR description:"ageing satisfaction" OR title:"attitudes towards own aging" OR subject:"attitudes towards own aging" OR description:"attitudes towards own aging" OR title:"attitudes towards own ageing" OR subject:"attitudes towards own ageing" OR description:"attitudes towards own ageing" OR title:"attitudes to ageing" OR subject:"attitudes to ageing" OR description:"attitud* to ageing" OR title:"attitudes to aging" OR subject:"attitudes to aging" OR description:"attitudes to aging" OR title:"aging attitudes" OR subject:"aging attitudes" OR description:"aging attitudes" OR title:"ageing attitudes" OR subject:"ageing attitudes" OR description:"ageing attitudes" OR title:"age attitudes" OR subject:"age attitudes" OR description:"age attitudes" OR title:"self directed aging stereotypes" OR subject:"self directed aging stereotypes" OR description:"self directed aging stereotypes" OR title:"self directed ageing stereotypes" OR subject:"self directed ageing stereotypes" OR description:"self directed ageing stereotypes" OR title:"aging stereotypes" OR subject:"aging stereotypes" OR description:"aging stereotypes" OR title:"ageing stereotypes" OR subject:"ageing stereotypes" OR description:"ageing stereotypes" OR title:"age stereotypes" OR subject:"age stereotypes" OR description:"age stereotypes" OR title:"age stereotype" OR subject:"age stereotype" OR description:"age stereotype"OR title:"awareness of aging" OR subject:"awareness of aging" OR description:"awareness of aging" OR title:"awareness of ageing" OR subject:"awareness of ageing" OR description:"awareness of ageing" OR description:"view on aging" OR title:"view on aging" OR subject:"view on aging" OR description:"views on aging" OR title:"views on aging" OR subject:"views on aging" OR description:"view on ageing" OR title:"view on ageing" OR subject:"view on ageing" OR description:"views on ageing" OR title:"views on ageing" OR subject:"views on ageing"title:"age based stereotypes" OR subject:"age based stereotypes" OR description:"age based stereotypes" OR title:"age beliefs" OR subject:"age beliefs" OR description:"age beliefs") AND (title:"cognitive control" OR subject:"cognitive control" OR description:"cognitive control" OR title:"cognition" OR subject:"cognitive" OR description:"cognitive" OR title:"cognition" OR subject:"cognition" OR description:"cognition" OR title:"cognitive decline" OR subject:"cognitive decline" OR description:"cognitive decline" OR title:"cognitive disorder" OR subject:"cognitive disorder")

Search 5

(title:"subjective age" OR subject:"subjective age" OR title:"subjective aging" OR subject:"subjective aging" OR title:"subjective ageing" OR subject:"subjective ageing" OR title:"felt age" OR subject:"felt age" OR title:"desired age" OR subject:"desired age" OR title:"perceived age" OR subject:"perceived age" OR description:"perceived age" OR title:"self perceptions of aging" OR subject:"self perceptions of aging" OR description:"self perceptions of aging" OR title:"self perceptions of ageing" OR subject:"self perceptions of ageing" OR description:"self perceptions of ageing" OR title:"perceptions of aging" OR subject:"perceptions of aging" OR description:"perceptions of aging" OR title:"perceptions of ageing" OR subject:"perceptions of ageing" OR description:"perceptions of ageing" OR title:"aging perceptions" OR subject:"aging perceptions" OR description:"aging perceptions" OR title:"ageing perceptions" OR subject:"ageing perceptions" OR description:"ageing perceptions" OR title:"age perceptions" OR subject:"age perceptions" OR description:"age perceptions" OR title:"personal experience of aging" OR subject:"personal experience of aging" OR description:"personal experience of aging" OR title:"personal experiences of aging" OR subject:"personal experiences of aging" OR description:"personal experiences of aging" OR title:"personal experience of ageing" OR subject:"personal experience of ageing" OR description:"personal experience of ageing" OR title:"personal experiences of ageing" OR subject:"personal experiences of ageing" OR description:"personal experiences of ageing"title:"aging related cognitions" OR subject:"aging related cognitions" OR description:"aging related cognitions" OR title:"aging cognitions" OR subject:"aging cognitions" OR description:"aging cognitions" OR title:"ageing related cognitions" OR subject:"ageing related cognitions" OR description:"ageing related cognitions" OR title:"ageing cognitions" OR subject:"ageing cognitions" OR description:"ageing cognitions" OR title:"age related cognitions" OR subject:"age related cognitions" OR description:"age related cognitions" OR title:"age cognitions" OR subject:"age cognitions" OR description:"age cognitions" OR title:"aging satisfaction" OR subject:"aging satisfaction" OR description:"aging satisfaction" OR title:"ageing satisfaction" OR subject:"ageing satisfaction" OR description:"ageing satisfaction" OR title:"attitudes towards own aging" OR subject:"attitudes towards own aging" OR description:"attitudes towards own aging" OR title:"attitudes towards own ageing" OR subject:"attitudes towards own ageing" OR description:"attitudes towards own ageing" OR title:"attitudes to ageing" OR subject:"attitudes to ageing" OR description:"attitud* to ageing" OR title:"attitudes to aging" OR subject:"attitudes to aging" OR description:"attitudes to aging" OR title:"aging attitudes" OR subject:"aging attitudes" OR description:"aging attitudes" OR title:"ageing attitudes" OR subject:"ageing attitudes" OR description:"ageing attitudes" OR title:"age attitudes" OR subject:"age attitudes" OR description:"age attitudes" OR title:"self directed aging stereotypes" OR subject:"self directed aging stereotypes" OR description:"self directed aging stereotypes" OR title:"self directed ageing stereotypes" OR subject:"self directed ageing stereotypes" OR description:"self directed ageing stereotypes" OR title:"aging stereotypes" OR subject:"aging stereotypes" OR description:"aging stereotypes" OR title:"ageing stereotypes" OR subject:"ageing stereotypes" OR description:"ageing stereotypes" OR title:"age stereotypes" OR subject:"age stereotypes" OR description:"age stereotypes" OR title:"age stereotype" OR subject:"age stereotype" OR description:"age stereotype"OR title:"awareness of aging" OR subject:"awareness of aging" OR description:"awareness of aging" OR title:"awareness of ageing" OR subject:"awareness of ageing" OR description:"awareness of ageing" OR description:"view on aging" OR title:"view on aging" OR subject:"view on aging" OR description:"views on aging" OR title:"views on aging" OR subject:"views on aging" OR description:"view on ageing" OR title:"view on ageing" OR subject:"view on ageing" OR description:"views on ageing" OR title:"views on ageing" OR subject:"views on ageing"title:"age based stereotypes" OR subject:"age based stereotypes" OR description:"age based stereotypes" OR title:"age beliefs" OR subject:"age beliefs" OR description:"age beliefs") AND (description:"cognitive disorder" OR title:"cognitive impairment" OR subject:"cognitive impairment" OR description:"cognitive impairment" OR title:"cognitive aging" OR subject:"cognitive aging" OR description:"cognitive aging" OR title:"dementia" OR subject:"dementia" OR description:"dementia" OR title:"Alzheimer" OR subject:"Alzheimer" OR description:"Alzheimer")

## Search strategy for DART

("Subjective age" OR "Subjective aging" OR "Subjective ageing" OR "age identity" OR "felt age" OR "desired age" OR "perceived age" OR "self-perceptions of aging" OR "self-perception* of ageing" OR "perception* of aging" OR "perception* of ageing" OR "aging perceptions" OR "ageing perception*" OR "age perception*" OR "personal experience* of aging" OR "personal experience* of ageing" OR "aging related cognition*" OR "ageing related cognition*" OR "age related cognition*" OR "aging cognition*" OR "ageing cognition*" OR "age cognition*" OR "aging satisfaction" OR "ageing satisfaction" OR "attitud* towards own aging" OR "attitud* towards own ageing" OR "attitud* to aging" OR "attitud* to ageing" OR "aging attitude" OR "aging attitude*" OR "ageing attitude*" OR "age attitud*" OR "self-directed aging stereotyp*" OR "self-directed ageing stereotyp*" OR "aging stereotyp*" OR "ageing stereotype*" OR "age stereotyp*" OR "awareness of aging" OR "awareness of ageing" OR "view* on aging" OR "view* on ageing" OR "age-based stereotyp*" OR "age belie*") AND (Attention OR memor* OR "executive funct*" OR "problem solv*" OR "process* speed" OR "speed of process*" OR "verbal fluency" OR naming OR multitask* OR inhibit* OR interference OR distract* OR "task switch*" OR reason* OR plan* OR intelligence OR "executive control" OR "cognitive control" OR cognit* OR "cognit* decline" OR "cognit* impairment" OR dementia OR alzheimer)
