# Supplementary material for: The Relationship between Subjective Aging and Cognition in Elderly People: A Systematic Review
Source: Healthcare (Basel). 2023 Dec 7;11(24):3115. doi: 10.3390/healthcare11243115 (PMC10743019; doi:10.3390/healthcare11243115)
Supplement: Supplementary file 1 [file healthcare-11-03115-s001.zip › healthcare-2727907-supplementary/Supplementary data 2.docx]

**Supplementary data 2**

*Quality assessment*

|  | Population representative | Same population | SA standarized | Cognition standarized | Relevant variables identified | Statiscally controlled | Numerical score | Overall quality |
| --- | --- | --- | --- | --- | --- | --- | --- | --- |
| Voelkner & Caskie (2023) | No | Yes | Yes | Yes | Yes | Yes | 5 | High |
| Stephan et al. (2023) | Yes | Yes | Yes | Yes | Yes | Yes | 6 | High |
| Langballe et al. (2023) | Yes | Yes | Yes | Yes | Yes | Yes | 6 | High |
| Levy & Slade (2023) | Yes | Yes | Yes | Yes | Yes | Yes | 6 | High |
| Chapman et al. (2022) | No | Yes | No | Yes | Yes | Yes | 4 | Moderate |
| Fernández-Jiménez et al (2022) | Yes | Yes | No | Yes | Yes | Yes | 5 | High |
| Sabatini et al (2022a) | Yes | Yes | Yes | Yes | Yes | Yes | 6 | High |
| McGarrigle et al. (2022) | Yes | Yes | Yes | Yes | Yes | Yes | 6 | High |
| Sabatini et al. (2022b) | Yes | Yes | Yes | Yes | Yes | Yes | 6 | High |
| Aftab et al. (2022) | Yes | Yes | Yes | Yes | Yes | Yes | 6 | High |
| Kaspar et al. (2022) | No | Yes | Yes | Yes | Yes | No | 4 | Moderate |
| Yuan et al. (2022) | No | Yes | Yes | Yes | Yes | Yes | 5 | High |
| Wahl et al. (2022) | Yes | Yes | Yes | Yes | Yes | Yes | 6 | High |
| Zhu & Neupert. (2021) | No | Yes | Yes | Yes | Yes | Yes | 5 | High |
| Sabatini et al. (2021) | Yes | Yes | Yes | Yes | Yes | Yes | 6 | High |
| Skoblow (2021) | Yes | Yes | Yes | Yes | Yes | Yes | 6 | High |
| Stephan et al (2021) | Yes | Yes | Yes | Yes | Yes | Yes | 6 | High |
| Morris et al. (2021) | Yes | Yes | Yes | Yes | Yes | Yes | 6 | High |
| Schönstein et al. (2021) | Yes | Yes | Yes | Yes | Yes | Yes | 6 | High |
| Mariano et al. (2021) | Yes | Yes | Yes | Yes | Yes | Yes | 6 | High |
| Qiao et al. (2021) | Yes | Yes | Yes | Yes | Yes | Yes | 6 | High |
| Stephan et al. (2021) | Yes | Yes | Yes | Yes | Yes | Yes | 6 | High |
| Kisvetrová et al. (2021) | No | Yes | Yes | Yes | Yes | Yes | 5 | High |
| Levy et al. (2020) | Yes | Yes | Yes | Yes | Yes | Yes | 6 | High |
| Wang et al. (2020) | Yes | Yes | Yes | Yes | Yes | No | 5 | High |
| Hajek et al. (2020) | Yes | Yes | Yes | Yes | Yes | Yes | 6 | High |
| Siebert et al. (2020) | Yes | Yes | Yes | Yes | Yes | Yes | 6 | High |
| Shao et al. (2020) | No | Yes | Yes | Yes | Yes | Yes | 5 | High |
| Cerino et al. (2020) | Yes | Yes | Yes | Yes | Yes | Yes | 6 | High |
| Choi et al. (2019) | No | Yes | Yes | Yes | Yes | Yes | 5 | High |
| Segel-Karpas & Palgi (2022) | Yes | Yes | Yes | Yes | Yes | Yes | 6 | High |
| Siebert et al. (2018) | Yes | Yes | Yes | Yes | Yes | Yes | 6 | High |
| Hughes & Lachman (2018) | Yes | Yes | Yes | Yes | Yes | Yes | 6 | High |
| Buggle (2018) | Yes | Yes | Yes | Yes | Yes | Yes | 6 | High |
| Levy et al. (2018) | Yes | Yes | Yes | Yes | Yes | Yes | 6 | High |
| Siebert et al. (2018) | Yes | Yes | Yes | Yes | Yes | Yes | 6 | High |
| Stephan et al. (2018) | Yes | Yes | Yes | Yes | Yes | Yes | 6 | High |
| Tyrrell (2017) | Yes | Yes | No | Yes | Yes | Yes | 5 | High |
| Seidler & Wolff (2017) | Yes | Yes | Yes | Yes | Yes | Yes | 6 | High |
| Stephan et al. (2017) | Yes | Yes | Yes | Yes | Yes | Yes | 6 | High |
| Jaconelli et al. (2017) | No | Yes | Yes | Yes | Yes | Yes | 5 | High |
| Robertson & Kenny (2016) | Yes | Yes | Yes | Yes | Yes | Yes | 6 | High |
| Robertson et al. (2016) | Yes | Yes | Yes | Yes | Yes | Yes | 6 | High |
| Jung (2016) | Yes | Yes | Yes | Yes | Yes | Yes | 6 | High |
| Stephan et al. (2016) | Yes | Yes | Yes | Yes | Yes | Yes | 6 | High |
| Hagood & Gruenewald (2015) | Yes | Yes | Yes | Yes | Yes | No | 5 | High |
| Hülür et al. (2015) | Yes | Yes | Yes | Yes | Yes | No | 5 | High |
| Ihira et al. (2015) | No | Yes | Yes | Yes | Yes | Yes | 5 | High |
| Chasteen et al. (2015) | No | Yes | Yes | Yes | Yes | No | 4 | Moderate |
| Shenkin et al. (2014) | Yes | Yes | Yes | Yes | Yes | Yes | 6 | High |
| Stephan et al. (2014). | Yes | Yes | Yes | Yes | Yes | Yes | 6 | High |
| Hughes (2014) | No | No | No | Yes | Yes | Yes | 3 | Moderate |
| Levy et al. (2012) | Yes | Yes | Yes | Yes | Yes | Yes | 6 | High |
| Sindi et al. (2012) | No | Yes | Yes | Yes | Yes | Yes | 5 | High |
| Trigg et al. (2012) | No | Yes | Yes | Yes | Yes | No | 4 | Moderate |
| Paggi et al. (2011) | Yes | Yes | Yes | Yes | Yes | No | 5 | High |
| Lee & Hong (2010) | No | Yes | Yes | Yes | Yes | Yes | 5 | High |
| Murphy (2009) | Yes | Yes | Yes | Yes | Yes | Yes | 6 | High |
| Levy & Langer (1994) | No | No | Yes | Yes | Yes | Yes | 4 | Moderate |
